# Willingness and associated factors of working with older people among undergraduate nursing students in China: a cross-sectional study

**DOI:** 10.1186/s12912-021-00639-7

**Published:** 2021-06-28

**Authors:** Yujie Guo, Lei Yang, Lingli Zhu, Yu Wan, Shujun Zhang, Jie Zhang

**Affiliations:** School of Nursing, Nantong University, Nantong City, Jiangsu Province China

**Keywords:** Older people, Willingness to care, Motivation, Undergraduate

## Abstract

**Background:**

The aging population has become a serious challenge for health care service and will lead to an increasing demand for nurses to work with older people. However, working with older people has always been an unpopular career choice among nursing students. This study aimed to further explore the willingness and associated factors of undergraduate nursing students to work with older people in China.

**Methods:**

A convenience sampling method was employed from May to July 2017 among undergraduate nursing students from a nursing school in Nantong China. Demographic data, the Chinese version of the Kogan’s Old Person’s Scale, the Chinese version of the Facts of Aging Quiz and the motivation questionnaire were used to collect data. A series of Mann-Whitney U test, Kruskal-Wallis H test, Spearman correlation test and Ordinal logistic regression analysis were applied to analyze the data.

**Results:**

Of the 853 students surveyed, 38.1 % were willing to work with older people after graduation. Expectancy, interest, attainment value, cost, prejudice, whether they like nursing profession and whether they participated in elderly-related activities were the most significant predictors of the students’ willingness to work with older people.

**Conclusions:**

Nursing students expressed a low level of willingness to work with older people upon graduation. Nursing educators have an important part in challenging students’ stereotype of older people and inspire their career choice in caring for older people through both well-designed curriculum and elderly-related activities, and relevant education departments should actively optimize aged-related courses, strengthen professional ethics and gratitude education, and improve nursing students’ sense of identity and mission in caring for older people, so as to improve their willingness to work with older people.

**Supplementary Information:**

The online version contains supplementary material available at 10.1186/s12912-021-00639-7.

## Background

An aging population has raised the public concern in the world, and has become a serious challenge for China. It is estimated that the number of people aged 65 or above in China has reached 158.31 million, which accounts for 11.4 % of the total population [1]. Aging is associated with health problems such as increasing diseases and functional disabilities. Most of the age-related problems are chronic, that require long term management [2]. It is estimated that the incidence rate of chronic diseases among older people in China is 75.1 % and the number of disabled and semi-disabled older people will continue to grow [3]. Therefore, as a key health care provider, there will be more possibilities for nurses, to care for older people in hospitals or communities, and meet the increasing demands placed on the health care system by an aging society. However, only 20,000 registered nurses have professional qualifications for gerontological nursing, while the demand is at least 150,000 [4], so there is a huge gap between supply and demand. It’s also worth noting that gerontological care is no longer just about focusing on old people’s life or disease, but also providing them with knowledge about disease prevention, physiotherapy, rehabilitation and health promotion, which requires nurses to be prepared with sufficient theoretical knowledge and practical skills in relation to the care of older people[5]. Thus, a growing number of registered nurses who are knowledgeable and committed to working with older people in different clinical settings are needed. Nursing students are the main source of nursing team to care for older people in the future, it is imperative to explore the willingness and influencing factors of nursing students to work with older people.

In recent years, some scholars have carried out research on the willingness of nursing students to work with older people. Rathnayake et al. reported in their study with a purposive sample of 98 that only 5.1 % of the respondents ranked older people as the first preferred group for their future career[6]. In the study of Mattos, only 1.6 % of the students indicated interest in pursuing gerontological nursing and students’ willingness to work with older people may be affected by their past experience of caring for older people or voluntary service[7]. Carlson and Idvall found that 44 % of students were reluctant to work with older people, indicating this work was slow-paced, depressing, boring and stressful, making nurses feel helpless[8]. A study conducted by Dobrowolska et al. showed that those who were not willing to work with older people were mainly due to the lack of aging knowledge, the bias against older people and inability to communicate with older people[9]. In Chi’s study, students’ positive attitudes about older people, paying attention to elderly-related issues, and having been a volunteer that served older people were predictors of their willingness to care for older people [10]. However, some studies reported although students had positive attitudes, they lacked interest in working with older people after graduation [11, 12]. In Li’s study, some students have a negative attitude towards older people, but they are still willing to choose careers related to older people under their own intrinsic motivation [13]. Thus it can be assumed that career motivation has a great influence on students’ willingness to choose their careers.

In most studies, students’ willingness to work with older people has been associated with age, gender, the experience of caring for older adults, the relationship with older people, knowledge about older people and attitudes towards older people and so on. The findings showed that these factors considerably impacted on nursing students’ career choice of working with older people [10, 14, 15]. However, career motivation as a potential influencing factor has not been effectively addressed. Career motivation is the reason for an individual to choose a certain profession, and it is the direct driving force for behavior. One way to explore the students’ motivation to choose to work with older people is through a lens of expectancy value theory. Expectancy-value theory identifies two key independent factors that influence behavior: the degree to which individuals believe they will be successful if they try (expectancy of success), and the degree to which they perceive that there is a personal importance, extrinsic value, intrinsic interest or cost in doing the task (task value) [16]. Specifically, task value includes: a given topic might be particularly interesting or enjoyable to the learner (interest or intrinsic value); learning about a topic or mastering a skill might be considered practical, or a necessary step toward a future goal (utility or extrinsic value); success in learning a skill might hold personal importance in its own right or as an affirmation of the learner’s self-concept (importance or attainment value); and focusing time and energy on one task means that other tasks are neglected (opportunity costs). The motivation questionnaire used in the current study was based on expectancy-value theory.

Therefore, this study aimed to take motivation into account and further explore the willingness and associated factors of undergraduate nursing students to work with older people, so as to provide some information for nurse educators to use in nursing education and development of curriculum. The effective development of career planning education and the reform of gerontological curriculum provide a basis for guiding nursing students to comply with social needs. This will help alleviate the practical problems of long-term care for older people in an aging society and improve the quality of elder care services.

## Methods

### Study Design, Setting and Sample

A descriptive cross-sectional study was employed from May to July 2017 among undergraduate nursing students from a nursing school in Nantong China. In China, nursing students must experience an eight to twelve months internship in a qualified hospital in the final year of the baccalaureate nursing program. Students from the first to fourth year enrolled in the undergraduate degree program were included in this study. The inclusion criteria were (1) enrolled in full-time undergraduate schools, (2) provided informed consent and volunteered for the study. The exclusion criteria were (1) suspended clinical practice, dropped out of school during the investigation or in poor health condition, and (2) refused to participate in the study. Finally, the questionnaire was distributed to 875 students invited and 853 questionnaires were successfully completed, giving a response rate of 97.37 %.

### Data collection tool

The self-fill questionnaire included three sections. The first was an introductory section explaining the purpose of the study and the option to fill out the questionnaire whilst assuring the anonymity of respondents. The second section collected demographic data on age, gender, grade, et al. The third section comprised of the Chinese version of the Kogan’s Old Person’s Scale (KAOP), the Chinese version of the Facts of Aging Quiz 1(FAQ1) and the Motivation questionnaire.

### Demographic data

Demographic data contain the following: age, gender, grade, place of residence, economic status, whether they were the only child at home, whether they lived with older family members, parents’ attitudes toward older people, whether they were cared by grandparents in childhood, relationships with grandparents, experience of taking care of older people, whether they like nursing profession, whether they participated in elderly-related activities. Further, one question was added and phrased as: Would you consider working with older people when you graduate? Students were instructed to answer Yes/Undecided/No.

### The Chinese version of the Kogan’s Old Person’s Scale (KAOP)

KAOP was a 34-item scale developed by Kogan consisted of 17 positive and 17 negative statements to evaluate the attitude towards older people [17]. It has two dimensions named KAOP+ (appreciation) and KAOP- (prejudice) [17]. Students were asked to respond on a 7-point likert scale ranging from “strongly disagree to strongly agree”. Total scale scores range from 34 to 238. Taking 136 as the median value, a higher average score indicated a more positive attitude toward older people [18]. The scale has been shown a great validity and reliability and widely used to evaluate the attitude towards older people. The instrument’s internal consistency measured by Cronbach’s alpha has been found to range from 0.66 to 0.77 for the positive subscale and 0.73–0.83 for the negative subscale [17]. The content validity index of the Turkish version was 0.94 and the Cronbach’s alpha was 0.84 for the total scale [19]. To the Italian version of KOAP, the content validity index was 0.81 and the overall Cronbach’s coefficient alpha was 0.76 [20], and in a Chinese study, the Cronbach’s α was tested 0.82 for the total scale (0.81 for Appreciation, 0.83 for Prejudice) [21].

### The Chinese version of the Facts of Aging Quiz 1(FAQ1)

FAQ 1 was used to assess student nurses’ knowledge about older people [22]. It consisted of 25 statements including physical, mental, and social aspects of older people with possible answers of “true”, “false” or “don’t know”. The total score ranged from 0 to 25, with higher scores reflecting greater knowledge about aging. The score calculated only correct answer and the option as “false” and “don’t know” were not scored. The Cronbach’s alpha value for Chinese version adapted by Wang et al. was 0.68 and the content validity was 0.82 [23]. FAQ1 tool was provided as attached file.

### The Motivation questionnaire

The motivation questionnaire based on the expectancy-value theory was in Chinese developed by Cheng and it was used to measure students’ motivation to choose gerontological nursing as a career [24]. This 20-item questionnaire was divided into an expectancy subscale and a value subscale. The expectancy subscale contained 6 items. The value subscale consisted of four dimensions (14 items), including interest (3 items), utility (3 items), importance (5 items) and cost (3 items). Each item is rated on a 5-point likert scale ranging from 1 (strongly disagree) to 5 (strongly agree). Higher scores indicate students possess higher expectancy and value in gerontological nursing. The Cronbach’s alpha value for two sub-scales was 0.83 and 0.87 [24].

### Data collection procedure

For students in Year 1 and Year 2, the questionnaires were distributed to the nursing students during the classroom after taking permission from the lecturer. The participants were told the purpose, the significance of the study, the outline of the study, the inclusion criteria, and the right to refuse participation in the study. Students who met the eligibility criteria and agreed to participate were given 20–30 min to complete the questionnaire and return it to the researcher. For students in Year 3 and Year 4, a web-link to the online questionnaire was disseminated to the potential students through we-chat group, the participants were also told the purpose and outline of the study via a participation information sheet at the first page of the online questionnaire and it was made clear that participation was to be voluntary.

### Ethical consideration

 The study was approved by the ethics committees of the university. It was made clear that participation was to be voluntary, participants’ names would not be used and confidentiality would be maintained by the researchers. Informed consent was obtained when they were willing to participate in the study.

### Statistical Analysis

Data were analyzed using IBM SPSS version 20.0. Descriptive statistical methods (percentage distributions, means and standard deviations) were used for data analysis. Mann-Whitney U test and Kruskal-Wallis H test were used to examine the group differences among participants. The Spearman correlation test was employed to evaluate the relationship between willingness and other rank variables. Ordinal logistic regression analysis was conducted to identify the factors influencing the willingness of nursing students to work with older people.

## Results

### Demographic Characteristics

Table 1 indicates the demographic characteristics of the participants. The average age was 21.41 ± 1.55 years. The sample consisted of 4.57 % males and 95.43 % females. 58.97 % of the participants was the only child at home, 74.33 % of the participants had a general economic status, 92.26 % of the participants lived with older family members and 85.82 of the participants had a good relationship with grandparents.

**Table 1:** Demographic characteristics of the nursing students (*N* = 853)

Characteristic	N(%)	Characteristic	N(%)
Gender		Parents’ attitudes toward older people	
Male	39(4.57)	Good	731(85.70)
Female	814(95.43)	General	120(14.07)
Grade		Worse	2(0.23)
Year 1	230(26.96)	Being cared by grandparents in childhood	
Year 2	198(23.21)	Yes	571(66.94)
Year 3	219(25.68)	No	282(33.06)
Year 4	206(24.15)	Relationship with grandparents	
Place of residence		Good	732(85.82)
Rural	502(58.85)	General	119(13.95)
Urban	351(41.15)	Worse	2(0.23)
Economic status		Experience of taking care of older people	
Good	25(2.93)	Yes	414(48.53)
General	634(74.33)	No	439(51.47)
Bad	194(22.74)	Like nursing profession	
Only child at home		Yes	393(46.07)
Yes	503(58.97)	No	460(53.93)
No	350(41.03)	Participated in elderly-related activities	
Live with older family members		Yes	493(57.80)
Yes	787(92.26)	No	360(42.20)
No	66(7.74)		

### Willingness to work with older people

Of the 853 students surveyed, 325 (38.1 %) were willing to work with older people after graduation, 411 (48.2 %) were unsure whether they would like to work with older people, and 117(13.7 %) were unwilling to work with older people.

### Attitudes towards older people

The total score of KAOP scale of nursing students ranged from 93 to 238 and the mean score was (159.18 ± 21.61), which was above the median value of 136, confirming relatively positive attitudes toward older people among the nursing students.

### Knowledge about older people

The score of FAQ1 scale of nursing students ranged from 0 to 22 and the mean score was (11.22 ± 3.34), which was at a low level in this part.

### Motivation to Choose Gerontological Nursing as a Career

The mean scores for nursing students’ expectancy and value aspects of gerontological nursing career motivation were (18.00 ± 3.63) and (42.79 ± 6.48), respectively. The scores of the four subscales of value were interest (3.28 ± 0.62), utility (3.01 ± 0.61), attainment value (2.93 ± 0.62) and cost (2.91 ± 0.68).

### Univariate Analysis of Nursing Students’ Willingness to Work with Older People

As shown in Table 2, gender, whether they lived with older family members, whether they were cared by grandparents in childhood, experience of taking care of older people, whether they like nursing profession, whether they participated in elderly-related activities, attitudes towards older people, knowledge about older people and career motivation toward gerontological nursing had a significant difference on students’ willingness to work with older people (*P* < 0.05), and it was positively correlated with their parents’ attitudes toward older people and relationship with grandparents, as indicated in Table 3.

**Table 2:** Comparisons of nursing students’ demographic variables and willingness to work with older people

Variables	Career Yes (*n* = 325)	Career Undecided (*n* = 411)	Career No (*n* = 117)	H-test/Z-test	*P*-value
Age, median (IQR)	22.00(3.00)	21.00(3.00)	21.00(3.00)	H = 4.598	0.100
Gender, n (%)				Z=-3.061	0.002^**^
Male	26(66.7)	8(20.5)	5(12.8)		
Female	299(36.7)	403(49.5)	112(13.8)		
Place of residence, n (%)				Z=-1.636	0.102
Rural	205(40.8)	229(45.6)	68(13.6)		
Urban	120(34.2)	182(51.8)	49(14.0)		
Only child at home, n (%)				Z=-0.238	0.812
Yes	191(38.0)	241(47.9)	71(14.1)		
No	134(38.3)	170(48.6)	46(13.1)		
Live with older family members, n (%)				Z=-2.338	0.019^*^
Yes	306(38.9)	380(48.3)	101(12.8)		
No	19(28.8)	31(47.0)	16(24.2)		
Being cared by grandparents in childhood, n (%)				Z=-2.625	0.009^**^
Yes	230(40.3)	276(48.3)	65(11.4)		
No	95(33.7)	135(47.9)	52(18.4)		
Experience of taking care of older people, n (%)				Z=-6.936	0.000^**^
Yes	201(48.5)	182(44.0)	31(7.5)		
No	124(28.2)	229(52.2)	86(19.6)		
Like nursing profession, n (%)				Z=-15.996	0.000^**^
Yes	256(65.1)	133(33.9)	4(1.0)		
No	69(15.0)	278(60.4)	113(24.6)		
Participated in elderly-related activities, n (%)				Z=-4.716	0.000^**^
Yes	221(44.8)	216(43.8)	56(11.4)		
No	104(28.9)	195(54.2)	61(16.9)		
Total KAOP, median (IQR)	165.00(28.50)	159.00(31.00)	151.00(34.00)	H = 35.998	0.000^**^
Prejudice, median (IQR)	48.00(17.00)	52.00(20.00)	57.00(22.00)	H = 30.832	0.000^**^
Appreciation, median (IQR)	80.00(21.00)	75.00(19.00)	73.00(19.00)	H = 23.032	0.000^**^
FAQ 1, median (IQR)	12.00(4.50)	11.00(4.00)	11.00(4.00)	H = 6.325	0.042^*^
Expectancy, median (IQR)	3.17(0.67)	3.00(0.50)	2.67(0.92)	H = 151.285	0.000^**^
Value, median (IQR)	3.29(0.57)	3.00(0.29)	2.64(0.71)	H = 210.529	0.000^**^
Interest, median (IQR)	3.67(1.00)	3.00(0.33)	3.00(0.33)	H = 192.421	0.000^**^
Utility, median (IQR)	2.61(0.50)	2.67(0.28)	2.89(0.50)	H = 106.892	0.000^**^
Attainment value, median (IQR)	3.00(0.60)	3.00(0.40)	2.20(1.00)	H = 145.984	0.000^**^
Cost, median (IQR)	3.00(1.00)	3.00(0.33)	3.00(0.67)	H = 33.055	0.000^**^

*IQR* Interquartile range (25–75 %), *KAOP* Kogan’s Old Person’s Scale, *FAQ1* Facts of Aging Quiz 1, *P < 0.05,**P < 0.01

**Table 3:** Correlation between demographic variables and students’ willingness to work with older people

Variables	Willingness to work with older people	
	r	*P*-value
Grade	− 0.053	0.123
Economic status	− 0.066	0.055
Parents’ attitudes toward older people	0.100	0.004^**^
Relationship with grandparents	0.110	0.001^**^

**P < 0.01

### Factors Affecting Nursing Students’ Willingness to Work with Older People

We used ordinal logistic regression analysis to identify factors associated with nursing students’ willingness to work with older people. Students’ willingness to work with older people was entered as the dependent variable, and variables that were significant in the results of the univariate analysis were entered as independent variables. Ordinal logistic regression showed that expectancy, interest, attainment value, cost, prejudice, whether they like nursing profession, whether they participated in elderly-related activities had a significant impact on the willingness of nursing students to work with older people (P < 0.05) (Table 4).

**Table 4:** Ordinal regression analysis of willingness to work with older people in undergraduate students

Variables	B	S.E	Waldχ2	*P*-value	95 %CI	
					Lower	Upper
Prejudice	− 0.015	0.006	7.082	0.008	− 0.026	− 0.004
Expectancy	0.090	0.029	9.529	0.002	0.033	0.147
Interest	0.179	0.059	9.288	0.002	0.064	0.294
Attainment value	0.166	0.041	16.789	0.000	0.087	0.246
Cost	− 0.141	0.040	12.289	0.000	− 0.219	− 0.062
Participated in elderly-related activities	0.326	0.154	4.469	0.035	0.024	0.628
Like nursing profession	1.923	0.179	115.345	0.000	1.572	2.274

## Discussion

### Willingness to work with older people

This study showed that 38.1 % of nursing students were willing to work with older people, 48.2 % were uncertain whether they were willing to work with older people, and 13.7 % were unwilling to work with older people. Most students are not clear about their willingness to work with older people, and they still hold a wait-and-see attitude. But the number of people who directly express that they are unwilling to work with older people is small, which indicates that in the context of aging, the booming aging industry had certain appeal to nursing students.

### Attitudes towards older people

This study showed nursing students had a positive attitude towards older people, which was congruence with the previous study by Hammar et al [25]. Attitude expresses an individual’s perception of someone, something, or a situation that may be related to an individual’s experience. Older people have richer knowledge, experience and wisdom that is worthy of our recognition. However, since older people tend to have low immunity and weakened mobility, most of them can only stay in bed and cannot take care of themselves well, this may increase the workload of the nurses. In addition, older people are prone to various psychological unhealthy manifestations such as anxiety, depression, loneliness, and emotional instability, which may affect the attitude of nursing students towards older people to some extent.

### Knowledge about older people

In this study, the FAQ1 score of nursing students was low and the pass rate was only 14.3 %. It is much lower than the pass rate of 21.83 % reported by Li [26], which indicated that the nursing students have poor mastery of aging knowledge and they lack the initiative and enthusiasm to learn aging knowledge. Therefore, it is necessary for educators to explore the content and methods of gerontological nursing, so that students can systematically grasp the relevant knowledge and improve their understanding of older people.

### Motivation to Choose Gerontological Nursing as a Career

The results showed students’ motivation to work with older people was at a moderate level, the scores of expectancy and value dimension were slightly lower than that of Cheng’s study [24]. Expectancy beliefs, or expectancies for success, are defined as how well one believes they will perform when engaging in a particular behavior in the near or distant future [27]. Tian et al. pointed out students with higher career expectations were more confident in facing problems [28]. The value of nursing profession for older people is personal interest, utility, attainment value and cost from strong to weak. Interest is the preference of nursing students for gerontological nursing and the fun they may get from it, reflecting the intrinsic value of gerontological work. Utility reflects students’ perceived benefits of gerontological care for their future development, such as salary and promotion opportunities, and is independent of the interest one has in working with older people. Attainment value is students’ perception of the importance of working with older people, reflecting the need for their self-fulfillment. Cost refers to the negative results stemming from an individual participating in the task, including costs that come directly from the task, as well as lost opportunities due to the time spent on this task[29]. Thus it could be seen interest is the highest value of gerontological work and students’ choice to work with older people may be related to the pursuit of their personal interests to a certain extent. Besides, it could be found nursing students haven’t realized the importance of caring for older people, and their understanding of this field is still limited. Older people tend to suffer from chronic diseases or disability which could be controlled rather than cured so that few students hope to make achievements and realize their life ideals in this field. The final is cost, which means the nursing students are in a wait-and-see attitude for the development of aged care and also uncertain about the possible costs of working with older people.

### Factors Affecting Nursing Students’ Willingness to Work with Older People

In this study, expectancy, interest, attainment value, cost, prejudice, whether they like nursing profession and whether they participated in elderly-related activities are the predictive factors of the nursing students’ willingness to work with older people.

### Perception of Nursing Profession

If a student didn’t like the nursing profession they were less likely to want to work with older people. Xie et al. pointed out in their study that liking the nursing profession and willing to get along with older people are the main potential resources in working with older people in nursing undergraduates [30]. The essence of nursing is caring, but the workload of nursing is heavy and the risks that need to be taken are large. Students who really love the nursing profession may have strong service conviction and dedication. At present, in the context of aging, the demand for nursing talents in aging-related fields is increasing. Therefore, students who love nursing profession are more willing to join this industry where elderly nursing talents are scarce.

### Participation in Elderly-Related Activities

Participation in elderly-related activities is a predictor of students’ willingness to work with older people. Koskinen et al. allowed students to communicate with older people on different topics through the Learning with Old People Programme and found that students in the intervention group were more interested in older people nursing [31]. Dickson et al. pointed out that students undertaking community practice learning were more likely to feel confident to take on community nursing roles [32]. This is because contact with older people can correct the one-sided understanding of the nursing staff on the older people and reverse their stereotype. Therefore, nursing educators should create more opportunities for students to contact with older older people and encourage them to communicate with older older people. Practical courses can be added in the teaching process, such as going to hospital, community, nursing home and other places to promote students’ understanding of older people.

### Attitudes towards older people

The more positive attitudes that nursing students have toward older people, the more willing they are to work with older people, consistent with other literature [33–35], which show if nursing students perceive the gerontological field in a positive light, they will be more likely to work with older people. The logistic regression suggested that nursing students’ prejudice attitudes toward older people were significantly predictive of their willingness to work with older people. Shen and Xiao also asserted prejudice was negatively associated with intention to work with older people [36]. Therefore, eliminating the negative attitude of nursing students to older people is an important task for nursing educators.

### Career Motivation

In logistic regression, expectancy, interest, attainment value, and cost were introduced into the equation. Expectation reflects the confidence of the nursing students in caring for older people, but the complexity of older people may require nurses to be equipped with the ability to improve their self-care agency, correctly identify the occurrence and development of older people’s disease, provide psychological care. One study have shown that lack of confidence will affect students’ willingness to work with older people, and the more confident the nursing students are, the more they can successfully cope with the problems that may arise in gerontological work [37].

Interest is the strongest motivation for nursing students to choose to work with older people. Pu et al. reported that nursing students who are interested in older people are more willing to work with older people [38]. At present, the choice of internship units in most schools is general hospital. The rehabilitation hospitals, nursing homes, and the community are rarely used as a base for internship, which makes nursing students have a one-sided understanding of older people and gerontological care. It is also difficult for students to combine the knowledge learned in class with practice, which greatly reduces the learning enthusiasm of nursing students.

The study also found that nursing students’ understanding of the importance of aged care is also an important predictor of their willingness to care for older people. This dimension reflects the need for self-realization. One study showed students’ career dreams were driven by eagerness to make a difference [39]. However, some students believe that gerontological nursing focuses on basic care and life care and they could hardly find a sense of accomplishment [40]. Therefore, educators should clarify the nurses’ role in gerontological care, so that nursing students can feel they can also make some achievements in taking care of older people.

Compared with general hospitals, there is still a huge gap in terms of techniques, medical resources and learning opportunities, which will limit nurses’ career development. The possible cost of working with older people is a consideration for nursing students. Utility failed to enter the regression equation, indicating that utility has no significant independent effect on the nursing students’ willingness to work with older people.

## Limitation

The large sample size available for this analysis of nursing students’ career intention was major strengths for this study. However, there are several limitations. First, this study is a single-center research, limiting the generalizability of the results. Second, the data analyzed were cross-sectional, and thus we were unable to comment on causal relationships and we couldn’t know the possible change of nursing students’ willingness to work with older people over time.

## Conclusions

Students expressed a low level of willingness to work with older people upon graduation. Although they held positive attitudes toward older people, there was a common lack of aging knowledge. The career motivation as an intrinsic force needs to be stressed. This suggests nursing educators have an important part in inspiring students’ career choice in working with older people and challenging students’ stereotype of older people through both well-designed curriculum and elderly-related activities. Besides, relevant education departments should actively optimize aged-related courses, strengthen professional ethics and gratitude education, and improve nursing students’ sense of identity and mission in working with older people, so as to improve their willingness to work with older people.

## Supplementary Information

**Additional file 1:**

## Data Availability

Data is available upon reasonable request from the corresponding author.

## References

[1] National Bureau of Statistics. Statistical Yearbook of China. 2018. http://www.stats.gov.cn/tjsj/ndsj/2018/indexch.htm. Accessed on 25 Apr 2020.

[2] Che CC, Chong MC, Noran NH. What influences student nurses’ intention to work with older people? A cross-sectional study[J]. Int J Nurs Stu, 2018: S0020748918301196. 10.1016/j.ijnurstu.2018.05.007 29852374

[3] Li YJ. Statue and influential factors of the elderly suffering from chronic diseases. Dissertation, North China University of Science and Technology, 2015.

[4] Cui HM, Sun Y, Duo R (2019). Research progress of nursing staff system abroad. Chin J Gerontol.

[5] Chen HJ, Chen JL (2015). Research progress on the training and education of professional elder nursing professionals. Chin J Nurs Educ.

[6] Rathnayake S, Athukorala Y, Siop S (2016). Attitudes toward and willingness to work with older people among undergraduate nursing students in a public university in Sri Lanka: A cross sectional study. Nurs Educ Today.

[7] Mattos M, Jiang Y, Seaman JB, Nilsen M, Chasens ER, Novosel LM (2015). Baccalaureate Nursing Students’ Knowledge of and Attitudes toward Older Adults. J Gerontol Nurs.

[8] Carlson E, Idvall E (2015). Who wants to work with older people? Swedish student nurses’ willingness to work in elderly care–a questionnaire study. Nurs Educ Today.

[9] Dobrowolska B, Jędrzejkiewicz B, Pilewskakozak A, et al. Age discrimination in healthcare institutions perceived by seniors and students. Nurs Ethics, 2017(1): 969733017718392. 10.1177/0969733017718392 28745574

[10] Chi MJ, Shyu ML, Wang SY, Chuang HC, Chuang YH (2016). Nursing Students’ Willingness to Care for Older Adults in Taiwan. J Nurs Scholarsh.

[11] King BJ, Roberts TJ, Bowers BJ (2013). Nursing Student Attitudes Toward and Preferences for Working with Older Adults. GerontolGeriatr Educ.

[12] Henderson J, Xiao L, Siegloff L, Kelton M, Paterson J. ‘Older people have lived their lives’: first year nursing students’ attitudes towards older people. Contemp Nurse. 2008;30(1):32–45. 10.5172/conu.673.30.1.32 19072189

[13] Li X. A research on implicit attitude to elderly people from college students, career choices of elderly related occupations, and the Relationship between them. Dissertation, Tianjin Normal University, 2016.

[14] Chai XH, Cheng C, Mei JJ, Fan XZ (2019). Student nurses’ career motivation toward gerontological nursing: A longitudinal study. Nurs educ today.

[15] Zhang S, Liu YH, Zhang HF, Meng LN, Liu PX (2016). Determinants of undergraduate nursing students’ care willingness towards the elderly in China: Attitudes, gratitude and knowledge. Nurs educ today.

[16] Cook DA, Artino AR (2016). Motivation to learn: an overview of contemporary theories. Med Educ.

[17] Kogan N (1961). Attitudes toward old people: the development of a scale and an examination of correlates. J Abnorm Soc Psycholo.

[18] Yang YJ, Li HP, Fang Q (2016). Investigation of Aging Knowledge and Attitude towards the Elderly among Five-year Higher Vocational Nursing Students. Nurs J Chin PLA.

[19] Erdemir F, Kav S, Citak EA, Hanoglu Z, Karahan A (2011). A Turkish version of Kogan’s attitude toward older people (KAOP) scale: Reliability and validity assessment. Arch Gerontol Geriat.

[20] Matarese M, Lommi M, Pedone C, Alvaro R, Marinis MGD (2013). Nursing student attitudes towards older people: validity and reliability of the Italian version of the Kogan Attitudes towards Older People scale. J Adv Nurs.

[21] Yen C, Liao W, Chen YM, Kao MC, Lee MC, Wang CC (2009). A Chinese version of Kogan’s Attitude toward Older People Scale: Reliability and validity assessment. Intern J Nurs Stud.

[22] Palmore E (1977). Facts on aging. A short quiz. Gerontologist.

[23] Wang CC, Liao WC, Kuo PC (2010). The Chinese version of the Facts on Aging Quiz scale: reliability and validity assessment. Intern J Nurs Stud.

[24] Cheng M. Factors influencing nursing graduates’ gerontological nursing career motivation. Dissertation, Shandong University, 2014.

[25] Hammar LM, Holmström IK, Skoglund K, Meranius MS, Sundler AJ (2017). The care of and communication with older people from the perspective of student nurses. A mixed method study. Nurs Educ Today.

[26] Li Y. An investigation on the attitude, knowledge and willingness of nursing students to the elderly. Dissertation, Jilin University, 2014.

[27] Kirk TN, Haegele JA. Expectancy-value beliefs, identity, and physical activity among adults with visual impairments. Disabil Rehabil. 2019:1–9. 10.1080/09638288.2019.1631395 31257943

[28] Tian Y, Cheng M, Cheng C, Fan XZ (2014). The impact of clinical learning environment and career motivation on career adaptability of student nurses. J Nurs Sci.

[29] Cheng C. Motivation towards gerontological caring and its associated factors among undergraduate nursing students: A longitudinal study. Dissertation, Shandong University, 2016.

[30] Xie H, Zhang J, Wang Q, Wang J, Hou PJ, Li JZ (2017). Willingness analysis of engaging in the elderly volunteer service and nursing geriatric in nursing undergraduates. J Bengbu Med Coll.

[31] Koskinen S, Salminen L, Puukka P, Leino-Kilpi H (2016). Learning with older people-Outcomes of a quasi-experimental study. Nurs Educ Today.

[32] Dickson CA, Morris G, Gable C (2015). Enhancing undergraduate community placements: a critical review of current literature. Br J Community Nurs.

[33] Nantan MB, Danino S, Freundlich N, Barda A, Yosef RM (2015). Intention of nursing students to work in geriatrics. Res Gerontol Nurs.

[34] Cheng M, Cheng C, Tian Y, Fan XZ (2015). Student nurses’ motivation to choose gerontological nursing as a career in China: a survey study. Nurs Educ Today.

[35] Haron Y, Levy S, Albagli M, Rotstein R, Riba S (2013). Why do nursing students not want to work in geriatric care? A national questionnaire survey. Int J Nurs Stud.

[36] Shen J, Xiao LD (2012). Factors affecting nursing students’ intention to work with older people in China. Nurs Educ Today.

[37] Yang YJ, Li HP, Fang Q (2016). A qualitative study on the willingness of five-year vocational nursing students to engage in elderly care. Vocational Technical Education.

[38] Pu LH, Hu XY, Liu ZY (2015). Investigation and analysis of attitude and knowledge towards elderly nursing among undergraduate nursing students. Chin J Mod Nurs.

[39] Øster I, Munk KP, Henriksen J. Career Dreams among Health Care Students: I Want to Make a Difference. Gerontol Geriatr Educ. 2017(3):1–14. 10.1080/02701960.2017.1311881 28350254

[40] Yin QL, Zhang XG, Lin L (2013). Qualitative study on nursing students’ willingness to take care of the elderly. Chin J Pract Nurs.

