# Supplementary material for: Willingness and associated factors of working with older people among undergraduate nursing students in China: a cross-sectional study
Source: BMC Nurs. 2021 Jun 28;20:113. doi: 10.1186/s12912-021-00639-7 (PMC8237413; doi:10.1186/s12912-021-00639-7)
Supplement: Supplementary file 1 — Additional file 1: [file 12912_2021_639_MOESM1_ESM.docx]

Appendix A. Palmore’s facts on aging quiz I

Directions: select the BEST response alternative for each of the questions below.

|  |  | True | False | Don’t know |
| --- | --- | --- | --- | --- |
| 1 | The majority of old people (age 65+) are senile (i.e. defective memory, disoriented, or demented) |  |  |  |
| 2 | All five senses tend to decline in old age. |  |  |  |
| 3 | Most old people have no interest in, or capacity for, sexual relations. |  |  |  |
| 4 | Lung capacity tends to decline in old age. |  |  |  |
| 5 | The majority of old people feel miserable most of the time. |  |  |  |
| 6 | Physical strength tends to decline in old age. |  |  |  |
| 7 | At least one-tenth of the aged are living in long-stay institutions (i.e. nursing homes, mental hospitals, homes for the aged, etc.). |  |  |  |
| 8 | Aged drivers have fewer accidents per person than drivers under age 65. |  |  |  |
| 9 | Most older workers cannot work as effectively as younger workers. |  |  |  |
| 10 | About 80% of the aged are healthy enough to carry out their normal activities. |  |  |  |
| 11 | Most old people are set in their ways and unable to change. |  |  |  |
| 12 | Old people usually take longer to learn something new. |  |  |  |
| 13 | It is almost impossible for most old people to learn new things. |  |  |  |
| 14 | The reaction time of most old people tends to be slower than reaction time of younger people. |  |  |  |
| 15 | In general, most old people are pretty much alike. |  |  |  |
| 16 | The majority of old people are seldom bored. |  |  |  |
| 17 | The majority of old people are socially isolated and lonely. |  |  |  |
| 18 | Older workers have fewer accidents than younger workers. |  |  |  |
| 19 | Over 15% of the population are now age 65 or over. |  |  |  |
| 20 | Most medical practitioners tend to give low priority to the aged. |  |  |  |
| 21 | The majority of older people have incomes below the poverty level (as defined by the Federal Government). |  |  |  |
| 22 | The majority of old people are working or would like to have some kind of work to do (including housework and volunteer work). |  |  |  |
| 23 | Older people tend to become more religious as they age. |  |  |  |
| 24 | The majority of old people are seldom, irritated or angry. |  |  |  |
| 25 | The health and socioeconomic status of older people (compared to younger people) in the year 2020 will probably be about the same as now. |  |  |  |
